# Methylene Blue Mitigates Doxorubicin-Induced Cardiotoxicity via KEAP1/NRF2/GPX-4/Caspase3 Modulation

**DOI:** 10.3390/ijms26167680

**Published:** 2025-08-08

**Authors:** Shaimaa G. Ibrahim, Ahmed M. Abu-Dief, Amany M. Gad, Enas S. Gad, Abdullah Yahya Abdullah Alzahrani, Alhafez M. Alraih, Ibrahim Omar Barnawi, Mona Mansour, Mohamed H. A. Gadelmawla, Ali Khames

**Affiliations:** 1 Department of Pharmacology and Toxicology, Faculty of Pharmacy, October 6 University, Giza 12585, Egypt; 2 Department of Chemistry, Faculty of Science, Sohag University, Sohag 82524, Egypt; 3 Department of Chemistry, College of Science, Taibah University, Madinah P.O. Box 344, Saudi Arabia; 4 Department of Pharmacology and Toxicology, Faculty of Pharmacy, Sinai University, Kantara Branch, Ismailia 41636, Egypt; 5 Department of Pharmacology, Egyptian Drug Authority (EDA)-Formerly NODCAR, Giza 12654, Egypt; 6 Department of Pharmaceutical Sciences, King Faisal University, Al-Ahsa 13889, Saudi Arabia; 7 Department of Chemistry, Faculty of Science, King Khalid University, Abha 61413, Saudi Arabia; 8 Department of Biological Sciences, Faculty of Science, Taibah University, Al-Madinah Al-Munawwarah 41321, Saudi Arabia; 9 Department of Pharmacology and Toxicology, Faculty of Pharmacy (Girls), Al-Azhar University, Nasr City, Cairo 11884, Egypt; 10 Department of Histology, Faculty of Dentistry, Sinai University, Kantara Branch, Ismailia 41636, Egypt; 11 Department of Pharmacology and Toxicology, Faculty of Pharmacy, Sohag University, Sohag 82511, Egypt

**Keywords:** doxorubicin, methylene blue, cardiotoxicity, oxidative stress, NRF2, troponin I, noradrenaline, apoptosis, p53, Caspase-3

## Abstract

Doxorubicin (Dox) is a potent anthracycline antitumor drug whose clinical utility is significantly restricted by its dose-dependent, cumulative cardiotoxicity, driven by increased oxidative stress, impaired antioxidant defenses, and apoptosis-mediated cardiomyocyte loss. Methylene blue (MB), a phenothiazine derivative with well-documented redox-modulating properties, is being explored as a viable cardioprotective agent due to its antioxidant and anti-apoptotic effects. This study evaluated the protective role of MB against Dox-induced cardiotoxicity in rats by examining its impact on oxidative stress markers (Kelch-like ECH-associated protein 1; KEAP1, nuclear factor erythroid 2-related factor 2; NRF2, Glutathione peroxidase 4; GPX-4, 8-hydroxy-2′-deoxyguanosine; 8-OHdG), neurohormonal indicators (noradrenaline), cardiac injury biomarkers (troponin I), and apoptotic mediators (p53, Caspase-3). Forty male albino rats were divided equally into four groups: control, Dox (15 mg/kg, i.p.), MB alone (4 mg/kg/day, p.o. for 7 days), and Dox plus MB. Dox administration significantly increased serum troponin I and noradrenaline levels, elevated cardiac KEAP1 and 8-OHdG, and reduced NFE2L2, NRF2, and GPX-4 expression. It also upregulated p53 and Caspase-3 and caused marked myocardial degeneration, necrosis, and inflammatory infiltration. MB co-treatment significantly reduced troponin I and noradrenaline levels, restored KEAP1/NFE2L2 (NRF2)/GPX-4 pathway balance, decreased oxidative DNA damage, and attenuated p53 and Caspase-3 activation, preserving myocardial architecture with minimal inflammatory changes. These findings demonstrate that MB confers potent cardioprotection against Dox-induced cardiac injury by enhancing antioxidant defenses, limiting oxidative DNA damage, suppressing apoptosis, and normalizing neurohormonal imbalance, suggesting its promise as an adjunctive strategy to mitigate anthracycline-associated cardiotoxicity.

## 1. Introduction

Doxorubicin (Dox) is widely recognized as a potent antineoplastic agent utilized for treating various types of cancer [1,2]. Effective utilization of Dox has been severely impeded by toxicities such as vomiting, anemia, nausea, extravasation, and hair loss; however, the most significant negative impact is cardiotoxicity [3]. The cardiotoxic effects of Dox are increasingly gaining interdisciplinary attention due to the rising number of cancer survivors. Dox-induced cardiomyopathy (DCM) is highly fatal, as it fails to respond to existing therapies and may present in either acute or chronic forms [4]. The mechanisms underlying DCM are complex and not yet fully understood. It should also be emphasized that the significance of many of the anticipated mechanisms remains to be established. Apoptosis-driven cardiomyocyte loss and oxidative stress, coupled with declined concentrations of sulfhydryl groups and antioxidants, are stated as the main cause of DCM [5]. Previous work indicates that NRF-2 has a potential impact in mitigating Dox-induced cardiotoxicity (DCT) and influencing mitochondrial biogenesis. NRF-2 deficiency has been linked to aggravated cardiac damage following acute Dox administration (25 mg/kg). In vitro studies show that NRF-2 reduced expression mirrors the cardiac muscle cell damage caused by Dox, while its overregulation decreases Dox toxicity by promoting scavenging ROS and autophagy [6]. Furthermore, Dox activates NFκB in endothelium [7] and the renal cells [8], initiating the transcription of inflammatory cytokines that may contribute to apoptosis [7,9]. Also, the downregulation of troponin-I and alteration of adrenergic function by Dox have been elucidated as a substantial mechanism of its cardiotoxicity [10]. Remarkably, Dox therapy decreased noradrenaline-induced contraction and α-adrenoceptor protein levels; however, the presence of superoxide dismutase (SOD), an enzyme that neutralizes ROS, partially reversed the decrease in α-adrenoceptor levels and restored some of the vessel contraction ability [11]. Increasing attention should be paid to the oxidative response to cardiomyopathy caused by Dox. Overall, these investigations revealed that preserving antioxidant status is a convenient way to reduce Dox’s cardiotoxic impacts.

Methylene blue (MB), a phenothiazine derivative, was selected for its prospective therapeutic impacts in many cardiovascular disorders [12]. Investigations have revealed that methylene blue can lessen oxidative stress and enhance cardiac function in animal models of cardiomyopathy [13]. MB is a strong inhibitor of NO synthase in vascular endothelium with diverse clinical applications [14], but is currently not Food and Drug Administration (FDA)-approved, with its use in life-threatening conditions remaining off-label [15]. MB can reduce inflammatory, oxidative, neurological, and myocardial injury throughout ischemia–reperfusion, including cardiac arrest, by acting as a free radical scavenger and preventing the conversion of oxygen into superoxide [16,17]. Many experimental investigations studied MB’s impact on biochemical pathways for treating vasoplegia [18], demonstrating better microvascular activity and reduced migration of inflammatory cells across the endothelium [19,20,21,22]. Beyond cardiomyopathy, MB maintains its preventive efficacy in in vivo models of stroke, optic neuropathy, and Parkinson’s disease [23,24]. MB also inhibits guanylate cyclase and enhances cardiac function and mean arterial pressure in septic shock by reducing cGMP and preventing smooth muscle relaxation in blood vessels. This leads to an improvement in arterial pressure and systemic vascular resistance [25]. Based on these multifaceted mechanisms, we rationalized that methylene blue could significantly alleviate the cardiotoxic of drugs like doxorubicin.

## 2. Results

### 2.1. MB Effects on Troponin-1 and Noradrenaline DCT

**Figure 1:**
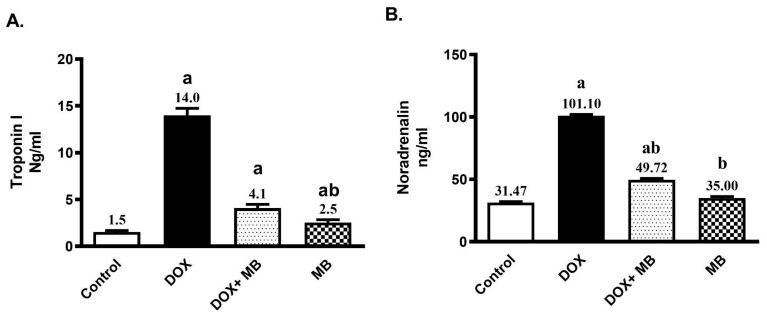
Effect of methylene blue (MB) on serum Troponin-1 (**A**), and Noradrenaline (**B**) values. Data are expressed as mean ± S.E.M (n = 6). MB was given at a dose (4 mg/kg/day, P.O.) for 7 days. Cardiac damage was induced by Doxorubicin (Dox) (15 mg/kg, IP, once. a: significantly different from the control group; b: significantly different from the Dox group at *p* < 0.05.

Troponin-1 and Noradrenaline serum values were substantially elevated in rats that received Dox (15 mg/kg, IP, once) by 8.1- and 2.2-fold, respectively, compared to the control (Figure 1). The group treated with MB (4 mg/kg/day, P.O. for 7 days) exhibited a remarkable decline in the concentrations of Troponin-1 and Noradrenaline by 70.9%, and 50.8%, respectively, compared to Dox (*p* < 0.05).

### 2.2. Effects of MB on Oxidative Stress Markers Against DCT

**Figure 2:**
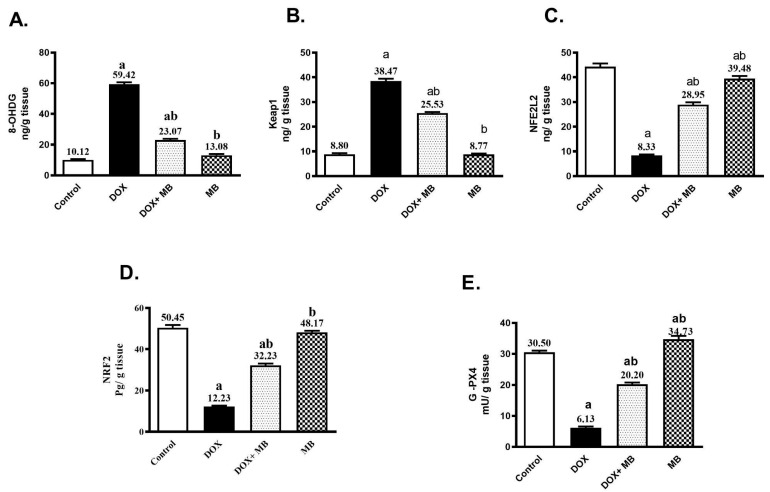
Effect of methylene blue (MB) on cardiac levels of (**A**) 8-hydroxy-2′-deoxyguanosine (8-OHdG), (**B**) Kelch-like ECH-associated protein 1 (KEAP1), (**C**) nuclear factor erythroid 2-like 2 (NFE2L2), (**D**) nuclear factor erythroid 2-related factor 2 (NRF2), and (**E**) glutathione peroxidase 4 (GPX-4) in rats. Data are expressed as mean ± S.E.M. (n = 6 per group). MB was administered orally at a dose of 4 mg/kg/day for 7 consecutive days. Cardiotoxicity was induced using a single intraperitoneal injection of doxorubicin (DOX, 15 mg/kg). a, significantly different from the control group; b, significantly different from the DOX group (*p* < 0.05).

The antioxidant effect of MB against ROS generation induced by Dox was assessed by estimating the contents of 8-OHdG, KEAP1, NFE2L2, NRF2, and GPX-4. The induction of Dox cardiotoxicity led to a significant increase in cardiac 8-OHdG and KEAP1 contents of about 4.9- and 3.4-fold, respectively, as well as a significant decrease in NFE2L2, NRF2, and GPX-4 by 81.2%, 75.8%, and 79.9%, respectively, compared to the control (Figure 2). Treatment with MB revealed a significant decline in the cardiac 8-OHdG and KEAP1 by 61.2%, and 33.6%, respectively. In addition, it reduced the decrease in NFE2L2, NRF2, and GPX-4 contents by about 247.4%, 163.5%, and 229.4%, respectively, when compared to Dox (*p* < 0.05).

### 2.3. Evaluation of Immunohistochemistry Changes in Caspase-3 and p53

**Figure 3:**
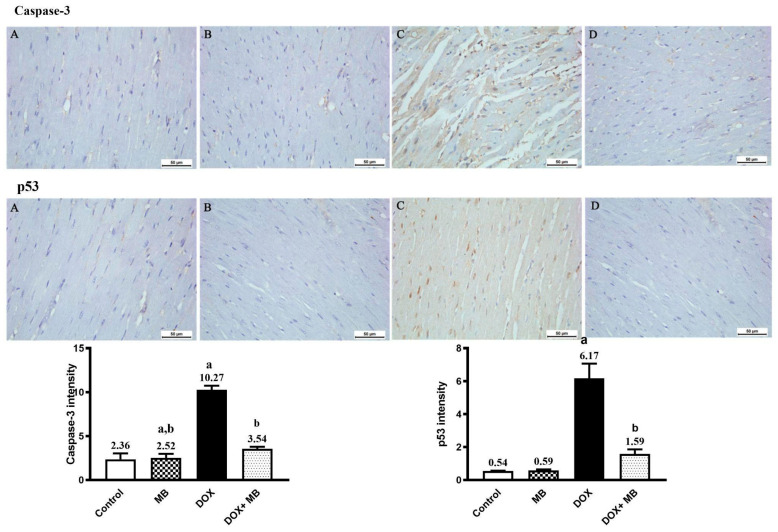
Photomicrographs of cardiac tissues showed anti-Caspase-3 and anti-p53 immunoreactivity. Both the control (**A**) and methylene blue (MB) (**B**) groups exhibited weak cytoplasmic Caspase-3 and p53 immunoreactivity. The doxorubicin (Dox) group (**C**) demonstrated moderate Caspase-3 and nuclear p53 immunoreactivity. The Dox+MB group (**D**) revealed weak, nearly normal Caspase-3 and p53 positivity. The histogram shows the results of a semi-quantitative IHC analysis. Values are mean ± SEM at *p* < 0.05. a: significance compared to control; b: significance compared to the Dox group.

IHC analysis showed a weak expression of Caspase-3 and p53 in cardiac tissues of the control and MB groups. The Dox group revealed moderate expression of p53 and Caspase-3. The treatment group (Dox+MB) revealed weak Caspase-3 and p53 immunoreactivity (Figure 3). There was substantial variance between the Dox group and treatment group regarding Caspase-3 and p53 IHC immunoreactivity (*p* < 0.01), with a remarkable decrease in Caspase-3 and p53 positivity in the treatment group (Figure 3).

### 2.4. Evaluation of Histological Changes

**Figure 4:**
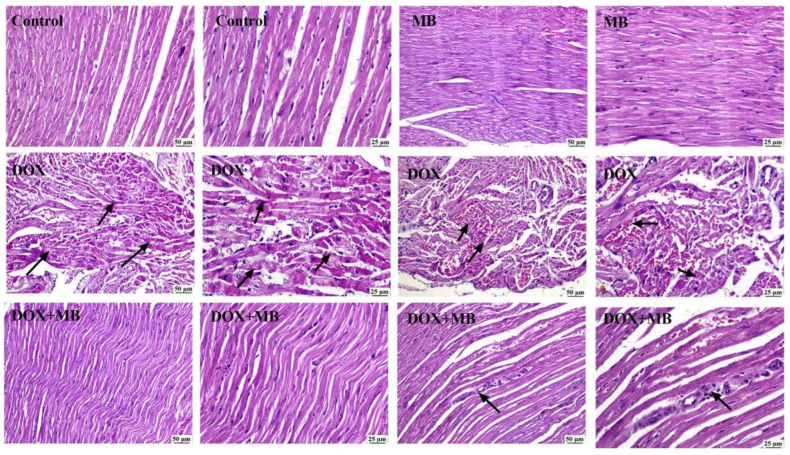
The photomicrographs illustrate the heart tissue from different experimental groups. In the control group, normal myocardium is observed at both standard and higher magnifications. The methylene blue (MB)-alone group shows normal myocardium at both magnifications. In contrast, the doxorubicin (Dox) group displays myocardial necrosis with fragmentation (arrows) and myocardial degeneration with fragmentation and hemorrhage (arrow) (H&E). Interestingly, the DOX+MB group exhibits normal myocardium at both standard and higher magnifications, with mild mononuclear inflammatory cell infiltration (arrow) also observed at both magnifications (H&E).

A microscopic evaluation of cardiac tissues of the normal control group (Figure 4) showed a normal myocardium structure. Likewise, the MB-alone group showed normal myocardium. The Dox group showed myocardial degeneration and necrosis, characterized by muscle hyalinization and fragmentation. Some of the examined sections showed mononuclear inflammatory cells’ infiltration and intermuscular hemorrhage. A marked improvement was noticed in the examined heart sections from the Dox+MB group, as they appeared normal except for a sporadic case that showed mild focal mononuclear inflammatory cells’ infiltration in-between myocardial fibers.

## 3. Discussion

Dox is a powerful and commonly used anticancer drug, with significant effectiveness against many kinds of solid tumors, including liver and breast carcinomas, in addition to hematological neoplasia [26]. Its indispensable role in modern oncology is clear; however, its clinical application is severely restricted by its dose-dependent, cumulative cardiotoxicity [27,28,29]. Alarmingly, the cardiac damage induced by doxorubicin can, in some cases, pose a greater risk to patients than the malignancy itself.

Dox’s cardiotoxic effects are well-documented in preclinical studies, where treated animals exhibit elevated serum levels of cardiac biomarkers such as troponin I, reflecting myocardial cell injury and the leakage of intracellular proteins due to cardiomyocyte membrane disruption [30,31]. Among the primary mechanisms implicated, oxidative stress is central. Dox undergoes redox cycling to form semiquinone free radicals, which react with molecular oxygen to produce ROS [32]. This oxidative cascade triggers lipid peroxidation, mitochondrial dysfunction, and irreversible damage to cardiomyocytes [33,34].

In the current study, we focused on the oxidative stress axis by evaluating the KEAP1/NFE2L2 (NRF2)/GPX-4 pathway and the oxidative DNA damage marker 8-OHdG, alongside noradrenaline and troponin I levels, as indicators of sympathetic activity and cardiac injury, respectively.

NRF2 is a master transcription factor regulating the cellular antioxidant response [35]. Under homeostatic conditions, NRF2 is bound in the cytoplasm by KEAP1, which promotes ubiquitination and eventual proteasomal destruction [36]. Under conditions of oxidative stress, KEAP1 causes structural modifications that activate NRF2, enabling it to enter the nucleus. There, NRF2 binds to antioxidant response elements (AREs) and upregulates the transcription of a battery of antioxidant and cytoprotective genes, including glutathione peroxidase 4 (GPX-4). GPX-4 plays a significant role in detoxifying lipid hydroperoxides and protecting cells from ferroptosis [37,38].

Dox administration in this study resulted in doxorubicin disturbing sympathetic regulation, as indicated by increased noradrenaline levels and which agrees with Vergaro et al. [39] and Minatoguchi [40]. Although sympathetic activation initially helps maintain cardiac output during stress or heart failure, excessive noradrenaline levels induced by doxorubicin can shift this response from compensatory to harmful, ultimately increasing cardiac workload and worsening dysfunction. [41,42,43,44]. Also, Doxorubicin treatment positively increases levels of troponin I, a highly sensitive and specific indicator of heart muscle injury; this finding is consistent with that of Drinković et al. [45] and Goje et al. [46]. When cardiomyocyte membranes are compromised, troponin I leaks into the circulation, signaling structural cardiac damage that can occur well before overt signs of heart failure develop [30]. The elevated troponin I levels detected in doxorubicin-treated rats in this study demonstrate the drug’s direct cardiotoxic impact at the cellular level. Sustained troponin elevation not only reflects ongoing myocardial cell loss but also serves as a robust predictor of future cardiac dysfunction in patients receiving anthracycline-based chemotherapy [47,48]. As such, tracking troponin I shows the severity of cardiac damage and supports the timely initiation of protective strategies.

In addition to these deleterious effects, significant disruption of this protective axis: KEAP1 levels were upregulated, while both NFE2L2/NRF2 expression and its downstream target GPX-4 were suppressed by doxorubicin, severely compromising the myocardium’s intrinsic defense against ROS. As a consequence, 8-OHdG was remarkably increased in cardiac tissue, indicating severe oxidative injury and genomic instability; this is compatible with Khan et al. [49], Abbas et al. [50], Liao et al. [51], and Kang et al. [52]. This finding aligns with the concept that excessive ROS generation overwhelms the endogenous antioxidant system, leading to peroxidation of lipids, proteins, and DNA damage. Doxorubicin disrupts the binding between KEAP1 and NRF2 by attaching to KEAP1, which in turn activates the NRF2 pathway and produces antioxidant enzymes as GPX4, showing cardioprotective effects [53].

Another mechanism by which doxorubicin causes cardiotoxicity is through apoptosis [54]. According to our findings, doxorubicin markedly elevated P53, a tumor-suppressor protein that detects DNA damage and triggers pro-apoptotic processes. In order to initiate programmed cell death, P53 then triggers Caspase-3, a crucial executioner Caspase that mediates the cleavage of cellular substrates; this is coincides with the findings of Wu et al. [55] and Khames et al. [56]. The loss of cardiomyocytes and compromised cardiac function were caused by extensive apoptosis in cardiac tissue, which was validated by elevated Caspase-3 levels.

We explored the cardioprotective potential of methylene blue to counteract these mechanisms.

In our study, methylene blue co-administration significantly mitigated DCT, as evidenced by a decrease in serum troponin I and noradrenaline, proving its cardioprotective effects through the maintenance of cardiac cell integrity and restoring cardiomyocyte survival [57,58].

Importantly, methylene blue reduced KEAP1 levels while upregulating NFE2L2/NRF2 and GPX-4, thereby restoring the heart’s antioxidant capacity and limiting lipid peroxidation and ferroptotic damage [59,60]. Methylene blue is A redox-active substance with proven antioxidant and mitochondrial protective qualities. It can prevent problematic parts of the electron transport chain by taking electrons from NADPH, which lowers ROS production at its source [61]. Interestingly, methylene blue also functions as an indirect activator of NRF2, increasing its nuclear translocation and the production of GPX-4 and other antioxidant enzymes. Accordingly, methylene blue significantly decreased 8-OHdG through its strong antioxidant effects [62].

Furthermore, when compared to the doxorubicin-only group, methylene blue significantly decreased P53 expression and Caspase-3 activation, indicating that it inhibited the pro-apoptotic response [63]. Methylene blue preserves cardiomyocyte viability by reducing DNA damage-induced apoptosis. Its protective effect stems from its ability to maintain mitochondrial integrity, which limits pro-apoptotic signaling and Caspase activation. MB downregulates p53 and Caspase-3, thereby interrupting the intrinsic apoptotic pathway and enhancing cell survival [64,65,66].

Interestingly, a histopathological examination confirmed these molecular findings: myocardial sections from animals treated with methylene blue plus Dox exhibited substantially preserved tissue architecture, minimal inflammatory infiltration, and reduced necrosis compared to the doxorubicin-only group, and this can be explained by methylene blue’s ability to reduce oxidative damage and apoptosis, retain cardiomyocyte membrane integrity, and restrict the recruitment of inflammatory cells and tissue degeneration.

**Figure 5:**
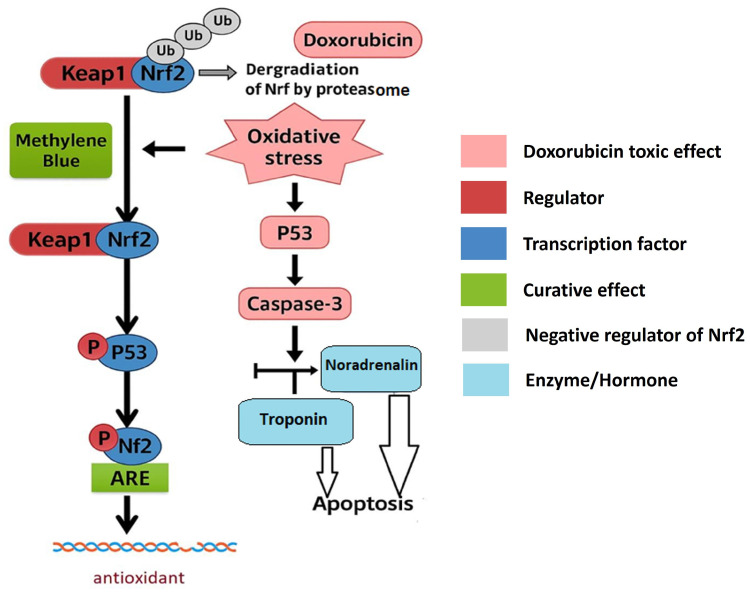
Summarized mechanism behind methylene blue’s cardioprotective effects.

The proposed mechanism behind the methylene blue cardioprotective effects, summarized in our accompanying schematic (Figure 5), highlights how methylene blue interrupts doxorubicin’s oxidative and apoptotic cascade by restoring the KEAP1/NFE2L2 (NRF2)/GPX-4 pathway, reducing oxidative DNA damage (8-OHdG), and suppressing the activation of P53 and Caspase-3. Additionally, the normalization of noradrenaline levels and restoration of cardiac cells’ integrity and function prevent cardiac remodeling due to decompensation under chemotherapeutic stress.

While our findings demonstrate the cardioprotective effects of methylene blue (MB) and its ability to modulate the KEAP1/NRF2/GPX-4 axis, a key limitation of the present study is the lack of direct evidence confirming NRF2 nuclear translocation. Although we observed a significant upregulation of NRF2 and its downstream effector GPX-4, these changes were assessed at the total protein expression level. Therefore, we cannot conclusively distinguish whether the observed NRF2 activation reflects increased nuclear translocation or cytosolic accumulation.

To definitively verify NRF2 nuclear activation, additional experiments, such as nuclear fractionation followed by Western blotting or immunofluorescence-based nuclear localization assays, would be necessary. Future studies incorporating these techniques are warranted to provide more direct mechanistic insight into MB’s regulation of NRF2 signaling. We acknowledge this as a limitation of the current work and encourage further exploration to elucidate the precise subcellular dynamics of NRF2 under MB treatment. This study lacks direct morphological and functional cardiac assessments, such as imaging or histology. As a result, the ability to correlate molecular changes with actual improvements in heart structure and performance remains limited.

## 4. Materials and Methods

### 4.1. Animals

Forty male albino rats weighing between 180 and 200 g were obtained from the animal house colony of NODCAR in Cairo, Egypt. Before the commencement of the experiments, the rats were acclimatized for 7 days. They were housed in stainless-steel cages under standard experimental conditions (temperature of 25 °C ± 2 °C, humidity at 50 ± 5%, and a 12/12 h light–dark cycle). The rats had free access to a standard chow diet and water. Moreover, the experiments were conducted after being assessed and approved by the Ethics Committee for Animal Experimentation at Sinai University [SU.REC.2024 (35 A)]. Our objective was to reduce animal distress throughout the duration of the experiment.

### 4.2. Drugs and Chemicals

Dox was supplied as doxorubicin hydrochloride (Sigma Chemical Co., St. Louis, MO, USA, CAS No. 25316-40-9), and MB was purchased from Sigma Chemical Co. (St. Louis, MO, USA, CAS No. 122965-43-9). All other chemicals employed in this study were of the utmost purity and analytical grade, and were sourced commercially.

### 4.3. Experimental Work

Animals (10 rats per group) were randomly allocated into 4 groups. Group I (Normal control) received a single intraperitoneal saline injection. Group II (Positive control): rats received a single dose of DOX (15 mg/kg; i.p. [67] to induce acute cardiomyopathy. Group III (drug control): rats were orally administered with MB (4 mg/kg/day) [68] for 7 consecutive days. Group IV (treatment): rats received a single dose of DOX (15 mg/kg; i.p.) and were orally administered MB after 1 h (4 mg/kg/day, P.O.) for 7 consecutive days

### 4.4. Blood Samples and Tissue Preparation

Twenty-four hours following the last administration of MB, blood samples were withdrawn from the retro-orbital plexus of the rats under light anesthesia to minimize pain and distress. The blood tubes were then left for 20 min before undergoing centrifugation at a speed of 3000 revolutions per minute for 15 min. Subsequently, the serum was isolated and preserved at −80 °C for later biochemical analysis. Following the blood sampling, the animals were euthanized via decapitation, and their heart tissues were collected. Approximately 50% of the heart tissues from each group were preserved in 10% formalin for histological and immunohistochemical examinations. The remaining heart tissues from the rats were stored at −80 °C for future assessment of parameters using the ELISA assay method.

### 4.5. The Histopathological Assessment

The cardiac tissues preserved in 10% formalin solution were subjected to histological assessment via staining with hematoxylin and eosin [69]. The histopathological analysis was conducted by a blinded histopathologist, who was unaware of the sample identities, to minimize potential errors.

### 4.6. Quantitative Assessment of the Rats’ Noradrenaline and Troponin I

Noradrenaline and troponin serum levels in rats were investigated using rat-specific ELISA kits (LSBIO, Seattle, WA, USA, Cat #: LS-F28027) and (My BioSource, San Diego, CA, USA, Cat #: MBS727624), respectively. The technique was processed as directed by the manufacturer’s instructions.

### 4.7. Determination of the Heart Tissue Concentrations of Oxidative Stress Markers: KEAP1, NFE2L2, NRF-2, GPX-4, and 8-OHdG in Rats

The heart tissue concentrations of KEAP1 (My BioSource, San Diego, CA, USA, Cat #: MBS7218529), NFE2L2 (LSBIO, Seattle, WA, USA, Cat #: LS-F12145), NRF-2 (My BioSource, San Diego, CA, USA, Cat #: MBS012148), GPX-4 (BT LAB, Shanghai, China, Cat #: E1787Ra) and 8-OHdG (My BioSource, San Diego, CA, USA, Cat #: MBS267513) were analyzed using rat specific ELISA assay kits. All procedures were carried out as directed by the manufacturer’s instructions.

### 4.8. Immunohistochemistry (IHC)

We used IHC as a useful method to investigate the location of the Caspase-3 and P53 proteins in heart sections, as well as their expression. Sections were dewaxed, rehydrated, and boiled in citrate buffer (pH 6.0) to remove antigens as part of these processes. The endogenous peroxidase activity in the tissue was quenched by applying 3% hydrogen peroxide for 20 min. Then, to prevent antibodies from attaching to tissue proteins in an unintended way, they were blocked with 5% bovine serum albumin (BSA). Sections (4 µm) were incubated with primary antibodies (90 min) [70]. The samples were then cleaned and exposed to horseradish peroxidase (HRP)-labeled secondary antibodies for 30 min. The target proteins were stained utilizing a diaminobenzidine (DAB) kit, and hematoxylin was employed for counterstaining. A digital imaging device connected to a light microscope (Leica, Flexacam i5) was used to take pictures of tissue sections. In all groups, area Caspase-3 and P53 immunoreactivity was assessed at ×400 magnification [71].

### 4.9. Quantitative Evaluation of IHC Staining

Protein localization and expression within tissues were studied using semi-quantitative immunohistochemistry (IHC), utilizing ImageJ Fiji software (version 1.2) for analysis and to perform deconvolution and downstream analysis [70].

### 4.10. Statistical Analysis

All data were collected, processed, and statistically evaluated using IBM SPSS, version 22.0 (IBM Corporation, Armonk, NY, USA). The results were presented as the mean and standard deviation (SD). The difference between groups was statistically assessed with GraphPad Prism 5 using one-way ANOVA, followed by Tukey’s Kramer Multiple Comparison Test. *p* values < 0.05 were considered significant

## 5. Conclusions

Methylene blue demonstrates significant cardioprotective potential against doxorubicin-induced toxicity. This protection is mediated through activation of the KEAP1/NRF2/GPX-4 antioxidant pathway, a reduction in oxidative DNA damage (8-OHdG), and the inhibition of apoptosis via the downregulation of p53 and Caspase-3. Notably, our study highlights a novel mechanism—methylene blue’s ability to normalize noradrenaline levels—thereby mitigating harmful neurohormonal overstimulation and preserving cardiac muscle function and viability.

## Data Availability

The data that support the findings of this study are available from the corresponding author upon reasonable request.
